# Influence of a Lordotic Cage Profile on Global and Segmental Lordosis in the Context of Lumbar TLIF Surgeries: A Retrospective Radiological Analysis

**DOI:** 10.3390/jcm13237012

**Published:** 2024-11-21

**Authors:** Steffen Schulz, Peter Fennema, Ali Darwich, Frederic Bludau, Marcus Rickert

**Affiliations:** 1 Orthopedic Trauma Surgery Center, University Medical Center Mannheim, Theodor-Kutzer-Ufer 1-3, 68167 Mannheim, Germany; ali.darwich@umm.de (A.D.); frederic.bludau@umm.de (F.B.); 2 AMR Advanced Medical Research, 8708 Männedorf, Switzerland; 3 Orthopädie Rickert, 63500 Seligenstadt, Germany; rickert@dr-marcus-rickert.de

**Keywords:** lumbar spine, transforaminal lumbar interbody fusion, lordotic cages, spinal sagittal balance

## Abstract

**Background/Objectives:** Cage implantation decompresses neural elements, stabilizes segments, and promotes fusion, with sagittal balance influenced by cage size, geometry, and position. This retrospective study compared the effects of lumbar interbody cages with 10° and 15° lordotic angles on global and segmental lordosis in patients undergoing transforaminal lumbar interbody fusion (TLIF). **Methods:** Data from 215 patients who underwent 259 TLIF procedures between 2018 and 2022 were analyzed. All the surgeries were performed by a single senior orthopedic spine surgeon, and cages were selected by the surgeon based on patients’ clinical and anatomical factors. Radiographic assessments included measurements of global and segmental lordosis. **Results:** Patients who received 15° cages demonstrated significantly greater segmental lordosis compared to those who received 10° cages in both bisegmental and monosegmental procedures (*p* < 0.001). While the global lordosis in the 10°-cage group remained unchanged postoperatively (*p* = 0.687), bisegmental procedures showed a small but statistically significant increase (*p* = 0.035). Moreover, global lordosis did not significantly differ between the 10°- and 15°-cage groups. **Conclusions:** Cage geometry significantly influenced segmental lordosis, with 15° cages achieving overall more superior radiographic results compared to 10° cages. However, global lordosis was unaffected by cage angle, thereby highlighting the multifaceted nature of factors that influence overall spinal alignment. These findings provide valuable insights into lumbar spine surgery, thus emphasizing the need for comprehensive preoperative planning and consideration of individual patient characteristics.

## 1. Introduction

Lumbar sagittal spine balance is a critical parameter for assessing the functional and biomechanical status of the lumbar spine and reflects the harmonious relationship among lumbar lordosis, pelvic incidence, and the sacral slope in the sagittal plane [1]. Lumbar sagittal spine balance is influenced by various factors, including age, sex, body mass index, and spinal pathology. Surgical interventions, such as lumbar interbody fusion with cage implantation, also play a significant role in this regard.

Cage implantation—a widely employed technique for treating lumbar disorders—aims to restore disc height, decompress neural elements, stabilize segments, and promote bony fusion. However, the alteration of lumbar sagittal spine balance resulting from cage implantation depends on various factors—primarily cage geometry, cage size, and cage position.

Cage geometry refers to the shape of the cage, which significantly influences segmental lordosis and translation. Rectangular cages yield less pronounced segmental lordosis and segmental translation, while trapezoidal or wedge-shaped cages produce more pronounced segmental lordosis and segmental translation [2]. Cage geometry also affects stress distribution, strain transfer at adjacent levels, and the risk of complications (such as subsidence or retropulsion).

With regard to cage size, the height and width of the cage impact disc space distraction, compression, and surrounding structures. Larger cages provide increased anterior column support but could elevate the risk of complications. Smaller cages reduce complications but could compromise stability and fusion rates.

With regard to cage position, the angle and location of the cage influence the balance, alignment, and sagittal parameters of the fused segment and the overall spine. Cage position also affects the contact area and pressure on vertebral endplates, thus influencing bone growth and fusion quality.

The preservation or restoration of sagittal balance has been identified as a crucial predictor of patient outcomes [3]. Consequently, lumbar sagittal spine balance is a critical consideration for cage implantation, influencing clinical outcomes and surgery complications. Optimal cage selection depends on individual patient characteristics; this necessitates careful preoperative planning and intraoperative assessment for successful lumbar fusion.

In the authors’ clinical practice, the 10° and 15° lordotic angles are common options for interbody cages, including the cages used in this study. These specific angles were selected due to their availability and frequent use in standard surgical procedures as well as the surgeon’s clinical experience and judgment. Evaluating outcomes associated with these angles provides a better understanding of their impact on lumbar lordosis in real-world scenarios.

Overall, the aim of this study is to evaluate the different effects of 10° and 15° lordotic lumbar cages on preoperative planning and intraoperative decision-making. Furthermore, this study explores the benefits and drawbacks of using 10° and 15° lordotic lumbar cages, thereby providing guidance for surgical selection. This study also examines the changes in global and segmental lumbar lordosis induced by the choice of lumbar cages, which enhances the comprehension of the influence of cage geometry on spinal alignment. The primary study outcomes include the assessment of postoperative global and segmental lumbar lordosis achieved with 10° and 15° titanium cages.

## 2. Materials and Methods

This study constitutes a retrospective comparative radiographic analysis of consecutive transforaminal lumbar interbody fusion (TLIF) procedures that employ either a 10° or a 15° kidney-shaped interbody cage, specifically the wedge-shaped titanium Roccia Multilif cages or Roccia Mini cages (Silony, Leinfelden-Echterdingen, Germany). All lumbar fusion procedures (L2–S1) conducted by a single senior orthopedic spine surgeon (MR) from January 2018 to October 2022 were retrospectively reviewed. This included 215 consecutive patients who had undergone 259 instrumented mono- or bisegmental TLIF during the specified timeframe. The same operative technique was uniformly applied to all patients, with the only variations being the implant systems used for interbody fusion.

The choice between a 10° cage or 15° cage for implantation was made through meticulous preoperative assessment by the surgeon, considering an individual patient’s anatomical variations, biomechanical factors, and clinical indications. While there was a general preference to use the 15° cage to achieve optimal lordotic correction, its use is associated with a higher risk of endplate subsidence, particularly in patients with less robust endplates. Therefore, in cases in which this perceived risk was a concern, the surgeon opted for a 10° cage. In contrast, for patients with sclerotic endplates, the 15° cage was more commonly selected, as these cages are generally less prone to subsidence.

The study’s exclusion criteria were those with revision surgeries for pseudarthrosis, infections (such as spondylodiscitis), and tumor diseases. Additionally, the use of implants other than those planned in the study, both in terms of lordosis and material, was considered an exclusionary factor.

The operated segments comprised L2–L3 in 13 patients, L3–L4 in 41 patients, L4–L5 in 158 patients, and L5–S1 in 106 patients.

All patients underwent three to six months of unsuccessful conservative treatment before surgery. The surgical approach involved a midline approach using a standardized technique. Patients were positioned in a standard neutral prone posture, and the TLIF procedure was conducted using a conventional open technique. Following spine exposure, a unilateral facetectomy was typically performed on the symptomatic side, accompanied by decompression through the resection of the ligamentum flavum. In addition, partial removal of the superior articular process was executed to enhance access to the disc space, achieve suitable angulation for cage insertion, and adequately decompress the neuroforamen.

During the temporary distraction, thorough disc space preparation was performed, which involved the removal of disc material and endplate preparation. Care was taken to avoid breaching the vertebral endplate during interbody preparation. The cleared disc space was filled with an autograft collected during decompression in both groups. Subsequently, the cage was anteriorly inserted—safeguarding neural structures—and positioned securely on the anterior cortical ring of the vertebral body. While a posterolateral fusion was excluded, the contralateral facet joint was opened, the contralateral lamina was decorticated, and then it was covered with bone graft. Only pre-bent rods were used, and no additional contouring or adjustments were made intraoperatively. Following screw insertion, bilateral compression force was used for the final tightening of rods and screws.

Postoperatively, patients were restricted by immediate ambulation motion restrictions, which included avoidance of forced flexing, bending, and twisting of the back, and heavy lifting for the initial three months after surgery. Notably, no specific recommendations were provided regarding the use of nonsteroidal anti-inflammatory drugs for postoperative pain.

**Figure 1:**
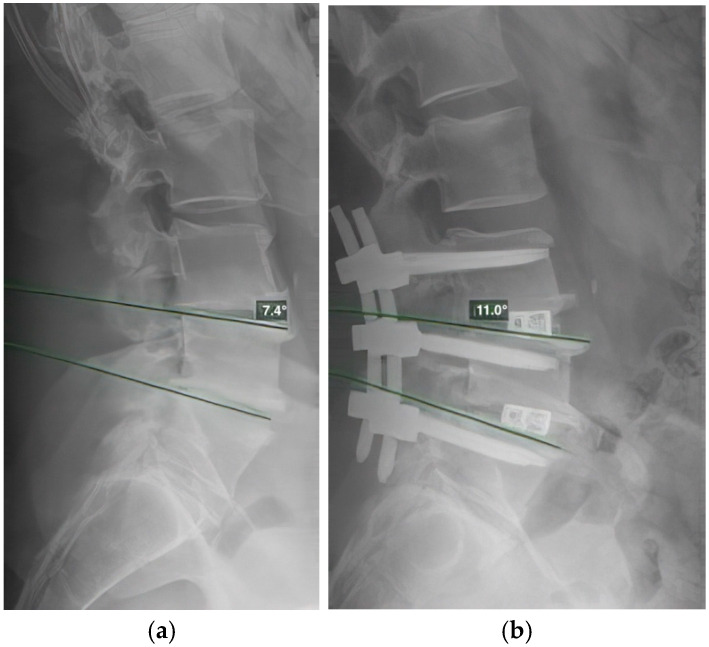
Measurement of segmental lordosis, defined as the angle between the tangent lines and the superior endplates of two adjacent vertebrae. (**a**): Lateral radiograph of the lumbar spine showing preoperative segmental lordosis and the measured segmental lordosis. (**b**): Lateral radiograph of the lumbar spine 6 months postoperatively, following spondylodesis of L3–L5 with 10° TLIF cages, showing the measured segmental lordosis of the L4–L5 segment.

To verify the placement of the interbody cages, we used radiographic assessment to confirm that the cages were positioned in the anterior third of the intervertebral space. Angular measurements included segmental lordosis, defined as the angle between tangent lines and the superior endplates of two adjacent vertebrae (Figure 1), with each segment measured separately in multisegmental procedures; and lumbar lordosis, defined as the angle between the tangent lines and the superior endplates of L1 and S1. All radiographic measurements were conducted at the post-operative six-month follow-up to assess global and segmental lumbar lordosis outcomes.

The study received approval from the Ethics Committee of the Medical Association of Hesse prior to its commencement (approval date: 15 July 2022; approval number: 2022-2918-evBO). All patients provided written informed consent. The study was performed in accordance with relevant guidelines and regulations, including the Declaration of Helsinki.

### Statistics

For continuous variables, the data were presented as mean ± standard deviation (SD); for categorical variables, the baseline data were presented as counts and percentages. Unpaired *t*-tests were used to assess the differences between the two cage types, while paired *t*-tests were employed to test differences between the preoperative and postoperative stages. The chi-squared test was used for binary variables. In addition, a linear regression analysis was conducted to explore the potential influence of lower lumbar segments on overall lumbar lordosis. Specifically, we included age, gender, and a variable that indicated whether the L5–S1 segment was involved in order to test the significance of these factors on lumbar lordosis outcomes. Two-sided *p*-values under 0.05 indicated statistical significance. Stata 15.1 software (Stata Corp., College Station, TX, USA) was used for statistical analysis.

## 3. Results

The clinical and radiological data of 215 patients who underwent 259 segmental procedures were available at baseline. Of these, 202 (78.0%) segments received a 10° cage, and 57 segments received a 15° cage. There were 167 monosegmental and 46 bisegmental procedures. Of the 46 bisegmental procedures, 6 exhibited discordant pairs (i.e., a 10° cage and a 15° cage). For the calculation of baseline characteristics, these patients were considered in both groups. The baseline characteristics of the study population are summarized in Table 1. The mean hospital stay was 8.3 ± 3.2 days in the 10°-cage group and 8.5 ± 3.5 days in the 15°-cage group (*p* = 0.712).

Radiography confirmed that all cages were correctly positioned in the anterior third of the intervertebral space. The mean lumbar segmental lordosis demonstrated a statistically significant increase from preoperative to postoperative for both the 10° cages and 15° cages in both the bisegmental and monosegmental procedures, with *p*-values < 0.001 in all instances (Table 2). Moreover, segmental lordosis was significantly greater in the 15° cages, both in the bisegmental and monosegmental procedures (*p* < 0.001, respectively).

Further, the mean lumbar global lordosis of the 10°-cage group exhibited no significant change between the preoperative and postoperative stages (*p* = 0.687); however, it revealed a small (i.e., 1.8°) but statistically significant increase from the preoperative stage to postoperative one for bisegmental procedures (*p* = 0.035). For the 15°-cage group, no significant differences from the preoperative stage to the postoperative one were observed (Table 2). Moreover, postoperative global lordosis did not significantly differ between the 10°- cage and 15°- cage groups. The linear regression analysis that was conducted to explore the influence of lower lumbar segments and baseline factors on overall lumbar lordosis revealed that the involvement of L5–S1 was not significantly associated with global alignment (*p* = 0.718). Additionally, neither age (*p* = 0.601) nor gender (*p* = 0.177) was significantly associated with global lumbar lordosis. No differences in duration of stay were found (*p* = 0.712).

### Postoperative Complications

Postoperative complications are listed in Table 3. There were 12 complications (5.9%) in the 10°-cage group and 6 (10.5%) in the 15°-cage group. However, there were no differences in terms of complication rates (*p* = 0.229). We observed cage subsidence in two cases in the 10° group (1.0%) and two cases in the 15°-cage group (3.5%). No statistically significant difference was found between the two groups (*p* = 0.211). Additionally, there were no instances of retropulsion noted in either of the cases.

## 4. Discussion

Improving fusion rates and reducing the incidence of adjacent segment disease have been associated with the restoration of spinal sagittal balance [3,4]. Attaining optimal sagittal alignment is the primary objective in fusion surgery; however, the precise postfusion sagittal plane contour of the lumbar spine remains unknown. The results from our study suggest that while the cage lordosis angle alone may not significantly influence global lordosis, segmental correction appears to be a critical factor in achieving optimal sagittal alignment. Emphasizing segmental lordosis correction in the lumbar spine segment targeted for fusion may be more effective in improving patient outcomes; this finding aligns with the existing literature [5]. This approach emphasizes the importance of prioritizing segmental alignment in surgical planning, particularly in cases in which achieving global alignment goals presents additional challenges. Variations in surgical technique, individual patient anatomy, and posterior instrumentation likely contribute to differences in alignment outcomes. Specifically, the use of TLIF procedures with a 10°- or 15° kidney-shaped interbody cage and pre-bent rods—performed by a single experienced surgeon—may likely have minimized the impact of cage angle on global lordosis.

Further, a decline in sagittal spinal curvature indexes following fusion surgery increases the likelihood of segmental breakdown both above and below the fusion level [5]. A more pronounced sagittal malalignment may even lead to iatrogenic flatback syndrome [6]. This significant sagittal imbalance results in a progressive failure of the dynamic and static stabilizers of the spine, thus causing pain and restricted functioning for the patient. Managing flatback deformity often necessitates intricate surgical restoration of sagittal balance, with clinical outcomes not always proving successful [5,6]. Our study provides a large-scale, retrospective analysis of the impact of cage geometry on global and segmental lordosis after TLIF. We found that the cage geometry had a significant effect on segmental lordosis, but not on global lordosis. The 15° cages achieved greater segmental lordosis than the 10° cages, regardless of the number of segments fused. However, global lordosis was not affected by the cage angle and only increased significantly in the bisegmental procedures with the 10° cages.

These results are consistent with previous studies that have revealed that cage lordosis, cage position, and surgical approach can influence segmental lordosis after TLIF [7,8,9,10]. However, our study also reveals a few novel findings that may have clinical implications. For example, we demonstrated that a higher cage lordosis (15°) can achieve better segmental correction than a lower cage lordosis (10°), regardless of the number of levels fused. This suggests that a higher cage lordosis may be preferable for achieving optimal segmental correction. This finding is in contrast with that of certain studies that have reported no significant differences between different cage lordosis angles [10,11] or even a negative correlation between cage lordosis and segmental lordosis [7]. This discrepancy may be due to the differences in the cage design, surgical technique, or patient selection. Additional studies are needed to compare the outcomes of different cage geometries and lordosis angles in a prospective and randomized manner.

We also showed that global lordosis, which is related to the overall spinal alignment and clinical outcomes, was not affected by cage lordosis. This indicates that other factors—such as the preoperative sagittal alignment, disc height, posterior instrumentation, and surgical technique—may play a more important role in determining the global lordosis after TLIF. Therefore, cage lordosis and geometry should not be considered in isolation but rather as part of a comprehensive surgical strategy that considers the individual patient’s anatomy and pathology. Previous studies have suggested that optimal global lordosis should be determined by pelvic incidence and that the segmental lordosis should be distributed proportionally among the lumbar levels [8]. However, the optimal distribution of segmental lordosis and the optimal cage geometry for each level remains unclear. More research is needed to investigate the long-term effects of cage lordosis and geometry on the clinical outcomes and adjacent segment degeneration after TLIF.

### Limitations

In this study, the nonrandomized allocation of patients to the study groups, based solely on the surgeon’s preference, introduces a potential limitation to the validity of the study. The absence of randomization raises the possibility that inherent differences in patient characteristics or disease severity between the two groups may have influenced the outcomes.

Furthermore, surgeon preferences, while informed by clinical judgment and experience, might introduce an element of bias and subjectivity into the group allocation process. To mitigate this limitation, future studies in this field may consider implementing randomized controlled trial designs in which the assignment of interventions is determined by chance, thereby minimizing selection bias and enhancing the robustness of the study’s conclusions. On the other hand, the group allocation—grounded in the surgeon’s expertise and judgment—was aimed at optimizing spinal alignment and stability, which contributed to the scientific rigor of the study by reflecting real-world surgical considerations.

## 5. Conclusions

In conclusion, our retrospective analysis of the impact of cage geometry on global and segmental lordosis after TLIF provides valuable insights into the nuances of lumbar spine surgery. The study revealed that cage geometry significantly influences segmental lordosis, with 15° cages achieving superior results compared to 10° cages, irrespective of the number of segments fused. However, global lordosis remained unaffected by the cage angle, thereby highlighting a disconnect between global and segmental alignment, which warrants further investigation. This discrepancy may stem from the complex interplay of multiple biomechanical factors, the full exploration of which was beyond the scope of our current study.

While our findings contribute to the existing body of knowledge, several questions remain unanswered. The optimal distribution of segmental lordosis and the most suitable cage geometry for each level require further exploration. Our study’s limitations, including nonrandomized group allocation and the potential for selection bias, emphasize the need for more rigorous investigations. Future studies, employing randomized controlled trial designs, could enhance the generalizability of our findings and provide a more robust foundation for guiding surgical decisions.

In essence, this study emphasizes the complexity of achieving optimal sagittal balance in lumbar fusion surgery. While cage geometry plays a pivotal role in segmental correction, a comprehensive understanding must include individual patient anatomy, pathology, and a broader surgical strategy. Thus, continued research in this field is essential to refine the surgical approaches, improve patient outcomes, and address the intricacies of adjacent segment degeneration.

## Figures and Tables

**Table 1:** The baseline characteristics of the study population.

	10° Cage (*n* = 202)	15° Cage (*n* = 57)	*p*-Value
Age	61.2 ± 12.4	57.4 ± 13.4	0.0463
Gender (male/female)	78:124 (38.6%:61.4%)	23:34 (40.4%:59.7%)	0.812
Level:			
L2–L3	3 (1.5%)	0 (0.0%)	0.085
L2–L4	8 (4.0%)	2 (3.5%)	
L3–L4	8 (4.0%)	5 (8.8%)	
L3–L5	18 (8.9%)	0 (0.0%)	
L4–L5	59 (29.1%)	14 (24.6%)	
L4–S1	48 (23.8%)	19 (33.3%)	
L5–S1	58 (28.7%)	17 (29.8%)	

**Table 2:** Restoration of preoperative lordosis (delta preoperative to postoperative).

Outcome	Statistic	Surgery Type	10° Cage (*n* = 202)	15° Cage (*n* = 57)	*p*-Value
Segmental lordosis [degrees]	Preoperative	Monosegmental	5.9 ± 4.0	5.8 ± 4.3	0.884
		Bisegmental	5.1 ± 3.3	5.9 ± 3.4	0.321
	Postoperative	Monosegmental	11.5 ± 2.5	13.7 ± 3.2	<0.001
		Bisegmental	10.4 ± 2.8	14.2 ± 1.9	<0.001
	Delta preoperative to postoperative (*p*-value)	Monosegmental	<0.001	<0.001	
		Bisegmental	<0.001	<0.001	
Postoperative global lordosis [degrees]	Preoperative	Monosegmental	50.9 ± 11.1	52.3 ± 15.9	0.528
		Bisegmental	47.9 ± 11.0	45.4 ± 11.6	0.371
	Postoperative	Monosegmental	50.5 ± 10.4	53.5 ± 11.7	0.137
		Bisegmental	49.7 ± 11.6	49.2 ± 14.1	0.876
	Delta preoperative to postoperative (*p*-value)	Monosegmental	0.687	0.452	
		Bisegmental	0.035	0.142	
Duration of stay [days]	Mean ± SD	Overall	8.3 ± 3.2	8.5 ± 3.5	0.712

All values are presented as mean ± standard deviation.

**Table 3:** Postoperative complications.

	10°-Cage Group (*n* = 202)	15°-Cage Group (*n* = 57)
Adjacent segment degeneration, requiring arthrodesis extension to L4–L5 at eight months	1	0
Cage intrusion, total	2	2
- Requiring cage revision/reoperation	1	0
- Not requiring cage revision/reoperation	1	2
MRSA Infection	2	0
Hematoma	3	0
Screw malpositioning	0	1
Wound infection/wound healing disturbance	4 *	3 *

* Two of these patients underwent bisegmental procedures that involved 10° cages and 15° cages, respectively.

## Data Availability

The datasets used and/or analyzed during the current study are available from the corresponding author upon reasonable request.
